# Remote monitoring of pacemakers and defibrillators: Effective and safe in Brazil?

**DOI:** 10.1016/j.hroo.2022.10.001

**Published:** 2022-12-16

**Authors:** Maria Eduarda Quidute Arrais Rocha, Neiberg de Alcantara Lima, Luís Gustavo Bastos Pinho, David Sales Pereira Gondim, Camila Pinto Cavalcante Miná, Eduardo Augusto Quidute Arrais Rocha, Maria Camila Timbó Rocha, Juvêncio Santos Nobre, Francisca Tatiana Moreira Pereira, Preeya Prakash, Fernanda Pimentel Arraes Maia, Eduardo Arrais Rocha

**Affiliations:** ∗ Department of Medicine, School of Medicine, University of Fortaleza, Fortaleza, Brazil; † Division of Cardiology, Department of Internal Medicine, Wayne State University, Detroit, Michigan; ‡ Department of Statistics and Applied Mathematics, Federal University of Ceara, Fortaleza, Brazil; § Postgraduate Program in Cardiovascular Sciences, Federal University of Ceara, Fortaleza, Brazil; ¶ Department of Medicine, School of Medicine, UNICHRISTUS, Fortaleza, Brazil

**Keywords:** Pacemaker, Implantable cardioverter-defibrillator, Remote monitoring, Middle-income countries, Brazil

## Abstract

**Background:**

The remote monitoring (RM) of cardiac implantable electronic devices (CIEDs) has become a common method of in-home monitoring and follow-up in high-income countries given its effectiveness, safety, convenience, and the possibility of early intervention. However, in Brazil, RM is still underutilized.

**Objectives:**

This observational study aims to demonstrate our experience of using RM in Brazil and the predictive factors of RM of CIED follow-up in Brazil.

**Methods:**

This was a prospective cohort study of patients with a CIED. Event rates are reported and clinical responses to those findings and outcomes based on the detection of RM. A logistic regression model was performed to identify predictors of more events, with *P* < .05 for statistical significance.

**Results:**

This study evaluated consecutive 119 patients: 30.2% with pacemakers, 42.8% with implantable cardioverter-defibrillator, 22.7% with cardiac resynchronization therapy (CRT) with defibrillator, and 3.3% with CRT with pacemaker. Events were detected in 63.9% of the cases in 29.5 ± 23 months of follow-up. The outcomes found were that 44.5% needed elective evaluation in medical treatment and 23.5% needed immediate evaluation in therapy. Logistic regression analysis showed that the groups with CRT or CRT with defibrillator (75.0%), reduced ejection fraction (76.5%), and New York Heart Association functional class ≥II (75.0%) had the highest RM event rates.

**Conclusions:**

RM proved to be effective and safe in the follow-up of patients with CIEDs in Brazil, allowing early interventions and facilitating therapeutic management.

**Key Findings**

▪ Remote monitoring proved to be effective in the follow-up of patients with cardiac implantable electronic devices, allowing early or elective interventions, which facilitated management of patients.
▪ The groups with cardiac resynchronization therapy or cardiac resynchronization therapy with defibrillator, reduced ejection fraction and most advanced New York Heart Association functional class had higher event rates.
▪ Remote monitoring should be considered as an additional form of follow-up in patients with cardiac implantable electronic devices in middle-income countries like Brazil.

## Introduction

The remote monitoring (RM) of cardiac implantable electronic devices (CIEDs) has become a common method of in-home monitoring and follow-up in high-income countries given its effectiveness, safety, convenience, and the possibility of early interventions.^1^, ^2^, ^3^, ^4^, ^5^

RM has shown reduction in morbidity and mortality. The early detection of alterations and the possibility of treatment even before the occurrence of symptoms has been the major advantage of this modality.^6^ Meanwhile, not every group of patients would benefit, there are costs associated with these devices, and a large number of recorded events may lead to a high physician burden.^7^, ^8^, ^9^

The COVID-19 pandemic revealed a great difficulty or even impossibility of ambulatory follow-up for several patients, especially those with heart disease and with a CIED. These patients need close monitoring for testing and programming their devices. Groups with RM could be followed during the pandemic from home.^10^, ^11^, ^12^

Brazil is a country of continental dimensions with a population exceeding 200 million people and with the largest economy in South America. Health care is a constitutional right since 1988, and is provided by both a national public and a private health system.^13^

As expected, the number of patients with CIEDs has increased over time; however, the specialists and health care facilities are still limited, so the country would benefit from new technologies to overcome this challenge.^14^

RM is still underutilized in Brazil. Despite having a favorable report, Brazilian regulatory agencies have not included RM in public or private networks; however, it has been used by some private centers and in research projects.^15^, ^16^, ^17^

This observational study aims to demonstrate our experience of using RM in Brazil and the predictive factors of RM of CIED follow-up in Brazil.

## Methods

### Study design

This was a prospective cohort study involving nonprobabilistic sampling of patients with RM in a private cardiology clinic after the implantation of a CIED and every 6 months after the procedure. The remote monitoring alert events were analyzed from all patients from November 2017 through March 2022.

### Remote monitoring

The RM systems used were from Biotronik (Lake Oswego, OR), with 3G transmissions via cell phone network, and from Abbott (Plymouth, MN), with transmissions via Internet modem, using the patient's network. Both systems used daily monitoring. The data were transmitted confidentially to the manufacturer centers and shared with the responsible team that consisted of 2 physicians with expertise in artificial cardiac stimulation and 2 technicians. Those messages were immediately checked after the e-mail alert was received. In the presence of any relevant findings, patients or family members were notified by telephone or text messages about the abnormalities as well as if any evaluations of the medical management were needed. In the absence of a reply text message, telephone calls were made. Events detected by the RM device were atrial and ventricular arrhythmias, delivered tachycardia therapies, battery or electrode alerts, and heart failure alerts. All the alert range for both manufacturer devices were maintained throughout the study period without programming changes.

Red alert transmissions were sustained ventricular or supraventricular arrhythmias, changes in lead impedance, generator battery depletion, and defibrillator therapies.

### Clinical data collected

The analyzed variables were age, sex, New York Heart Association (NYHA) functional class ≥II, ejection fraction (EF), type of CIED (ie, pacemaker, implantable cardioverter-defibrillator [ICD], or cardiac resynchronization therapy with defibrillator [CRT-D] or CRT with pacemaker), and presence of red alert transmission.

### Primary outcomes

Two different outcomes were studied: if the detected episode would require elective therapy evaluation or urgent therapy evaluation.

Elective therapy evaluation was defined as medical consultations that were scheduled with the specialist within 1 week of the alert, if the phone call or message had not resolved the problem. These alerts were atrial tachycardia (AT) or atrial fibrillation (AF) or nonsustained ventricular tachycardia (VT), sensitivity changes as atrial sensing amplitude <0.5 mV or ventricular sensing amplitude<2 mV, biventricular pacing <85% of the time, elective replacement indicator indicating battery depletion, and reduction in thoracic impedance referring to the heart failure algorithm that estimates thoracic fluid exceeding the manufacturer programmed threshold. The difference in thoracic impedance is part of the manufacturer (Abbott) settings and is based on a drop in the average of the prior 2 weeks.

Urgent therapy evaluation was defined as medical consultations that were scheduled with the specialist immediately, if after the phone call or message the issue had not resolved. Some patients were referred to an emergency room. These alerts were ventricular therapy episodes, ICD therapy disabled, backup mode activated (reset mode), battery at end of life, significant change in shock impedance (<30 Ω or >150 Ω), or mean AT or AF >130 beats/min for more than 10% of the day.

### Secondary outcome

We also analyzed the physician and patient overall sense of safety using RM by a simple question (“Did you feel safer using the RM?”). We also asked the physicians if they had an increase in workload . We compared the difference in events between patients who lived in the city of our hospital (Fortaleza, Brazil) and who lived outside the city.

### Statistical analysis

The statistical analysis used was logistic regression models, with *P* < .05 for statistical significance. The explanatory variables were selected by a stepwise selection method based on the Akaike information criterion as a measure to choose the best explanation model.

The analyzes between the groups were done using the chi-square test and paired Student *t* tests.

## Results

This study evaluated 119 patients with a mean age 72 ± 14.2 years; median ejection fraction was 55% (interquartile range, 34.7%–57%), 57.1% were in NYHA functional class ≥II, 30.2% had single or dual chamber pacemakers, 42.8% had ICDs, 22.7% had CRT-D, and 3.3% had CRT with pacemaker. A total of 75% of the patients lived in the city and 25% lived in the countryside (Table 1).

**Table 1:** Baseline characteristics of patients (N = 119)

Age, y	72.3 ± 14.3
Diabetes mellitus	40 (33.6)
Ejection fraction, %	55 (34.7–57.0)
Ejection fraction <35%	34 (28.5)
Hypertension	34 (28.5)
Stroke	23 (19.3)
Atherosclerotic disease	70 (41.1)
NYHA functional class ≥II	68 (57.1)
PM	36 (30.2)
ICD	51 (42.8)
CRT-D	27 (22.7)
CRT	4 (3.4)
Live in countryside	30 (25.2)
Follow-up, mo	29.5 ± 23

Values are mean ± SD, n (%), or median (interquartile range).

CRT = cardiac resynchronization therapy; CRT-D = cardiac resynchronization therapy with defibrillator; ICD = implantable cardioverter-defibrillator; NYHA = New York Heart Association; PM = dual chamber pacemaker.

### CIED RM events

Events were detected in 63.9% of the 119 patients’ RM interrogations over 29.5 ± 23 months of follow-up; 36.1% had no events. Of those with an event, 27.7% had 2 or more events detected and 16% had more than 6 events. The most common events were 18.5% with alterations in the combined outcomes of electrode impedance changes or battery depletion alerts or changes in the thoracic impedance parameters, 33.6% with sustained and nonsustained VT, 15.9% with sustained VT, and 44.5% with supraventricular arrhythmias (Table 2).

**Table 2:** Remote monitoring–detected events

Total events	76 (63.9)
2 or more events	27 (27.7)
More than 6 events	19 (16.0)
Ventricular arrhythmia†	40 (33.6)
Sustained ventricular tachycardia	19 (15.9)
ICD shocks‡	13 (10.8)
Supraventricular arrhythmia§	53 (44.5)
AF	39 (32.8)
HARE	37 (31.1)

Values are n (%).

AF = atrial fibrillation; HARE = high atrial rate episodes; ICD = implantable cardioverter-defibrillator.

† Sustained and nonsustained ventricular tachycardia.

‡ Some patients had more than 1 shock in the same episode of the remote monitoring.

§ HARE and AF.

### Primary outcomes

**Figure 1:**
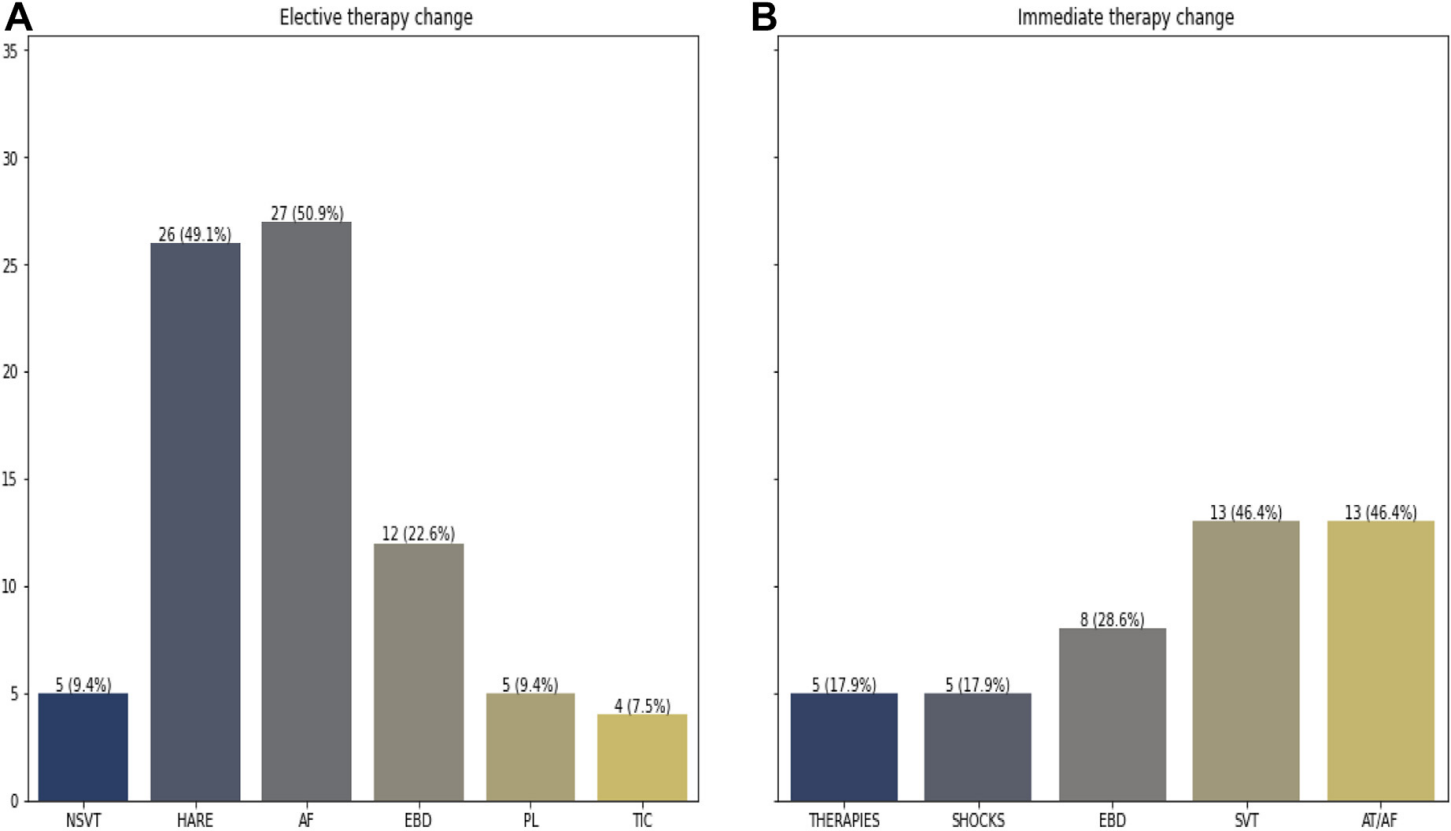
**A:** Elective therapy evaluation with remote monitoring. EBD indicates that the electrode or battery includes ventricular sensing amplitude 2 mV, atrial sensing amplitude <0.5 mV, and elective replacement time; PL indicates biventricular pacing <85%; and TIC indicates changes in mean thoracic impedance over the last 13 days (as determined by the manufacturer). **B:** Immediate therapy evaluation with remote monitoring. Therapies include atrial tachycardia (AT) pacing therapy and shocks; shocks include appropriate and inappropriate shocks of implantable cardioverter-defibrillator. EBD indicates that the electrode and battery include implantable cardioverter-defibrillator therapy disable, reset mode, end-of-life battery, and change in impedance shocks. AT and AF refer to a patient experiencing >130 beats/min more than 10% of the day (as determined by the manufacturer). AF = atrial fibrillation; HARE = high atrial rate episodes; NSVT = nonsustained ventricular tachycardia; SVT = sustained ventricular tachycardia.

The outcomes found were that 44.5% needed elective therapy evaluation in medical treatment and 23.5% needed urgent therapy evaluation (Figure 1A and 1B)

The groups with CRT or CRT-D (75.0%), reduced EF (76.5%), and NYHA functional class ≥Il (75.0%) had the highest event rates.

A red alert transmission occurred in a total of 23 (19.3%) patients. This variable was statistically significant to the 2 outcomes: elective therapy evaluation (*P* = .048) and urgent therapy evaluation (*P* = .007). The presence of sustained VT was statistically significant in 1 outcome, with a *P* value of .039 (elective therapy evaluation). NYHA functional class ≥II was associated with the urgent therapy evaluation (*P* = .047) (Tables 3 and 4).

**Table 3:** Logistic regression analysis and elective therapy evaluation

Variable	OR	95% CI		*P* Value
		Lower	Upper	
Age	0.9890	0.9600	1.0189	.553
Sex	0.8165	0.3239	2.0582	.779
CHF	1.5042	0.3664	6.1755	.536
DM	0.4890	0.1927	1.2411	.799
Stroke	0.5010	0.1645	1.5257	.122
NYHA functional class >II	0.5846	0.2872	1.1903	.061
CIED	1.0039	0.6100	1.6523	.800
EF	0.9918	0.9440	1.0419	.603
Events				
AF	2.6949	0.8981	8.0864	.082
HARE	1.8839	0.6054	5.8625	.217
SVT	0.4920	0.0987	2.4529	.039
Red alerts	5.8180	1.1971	28.2754	.048

EF was analyzed as a continuous variable. Sex, CHF, DM, stroke, and NYHA functional class >II were analyzed as dichotomous variables.

AF = atrial fibrillation; CHF = congestive heart failure; CI = confidence interval; CIED = cardiac implantable electronic device; DM = diabetes mellitus; EF = ejection fraction; HARE = high atrial rate episodes; NYHA = New York Heart Association; OR = odds ratio; SVT = sustained ventricular tachycardia.

**Table 4:** Logistic regression analysis and urgent therapy evaluation

Variable	OR	95% CI		*P* Value
		Upper	Lower	
Age	0.9665	0.9295	1.0049	.087
Sex	0.8638	0.2608	2.8616	.811
CHF	0.1358	0.0179	1.0310	.054
DM	0.7715	0.2509	2.3722	.651
Stroke	1.2275	0.3394	4.4400	.755
NYHA functional lass >II	0.3938	0.1570	0.9875	.047
CIED	0.7999	0.4293	1.4905	.482
EF	1.0066	0.9452	1.0721	.837
Events				
AF	2.0968	0.5312	8.2763	.291
HARE	1.9005	0.4479	8.0633	.384
SVT	3.4019	0.6787	17.0512	.137
Red alerts	10.4874	1.9257	57.1138	.007

EF was analyzed as a continuous variable. Sex, CHF, DM, stroke, and NYHA functional class >II were analyzed as dichotomous variables.

AF = atrial fibrillation; CHF = congestive heart failure; CI = confidence interval; CIED = cardiac implantable electronic device; DM = diabetes mellitus; EF = ejection fraction; HARE = high atrial rate episodes; NYHA = New York Heart Association; OR = odds ratio; SVT = sustained ventricular tachycardia.

### Secondary outcomes

Results from the survey showed that 86.5% of patients and 91.6% of physicians reported feeling safer using RM. Because 16% of the total cases had a large number of transmissions (>6 events), 10.9% of physicians reported a substantial increase in workload. All patients and physicians answered the questions.

### Additional outcomes

A total of 26 (21.8%) patients died during the follow-up period of 29.5 ± 23 months, with 5% due to COVID-19, and these patients did not have their RM transmission devices in the hospitals when they passed away due to the prohibition of carrying these equipment in the units. Twenty (16.9%) patients needed to start anticoagulation.

There was a 21.8% dropout rate due to financial, patient request, or logistic reasons (ie, lack of Internet connection), while 10.9% of technical problems that needed corrections were detected.

There was no difference in the number of events between those who lived in the city or in the countryside.

## Discussion

### Efficacy of RM in Brazil

This study presents data of this observational report of a successful RM program in Brazil. The data confirm the observations previously described in other countries in which RM is routinely performed. Our findings also contribute to the selection of groups that may have greater benefit from RM, such as patients with CRT or CRT-D, ventricular dysfunction, or heart failure.

The high number of events monitored, even during the COVID-19 pandemic, a time when patients did not have broad access to health care facilities, reinforce the importance of having out-of-hospital monitoring in our country.

**Figure 2:**
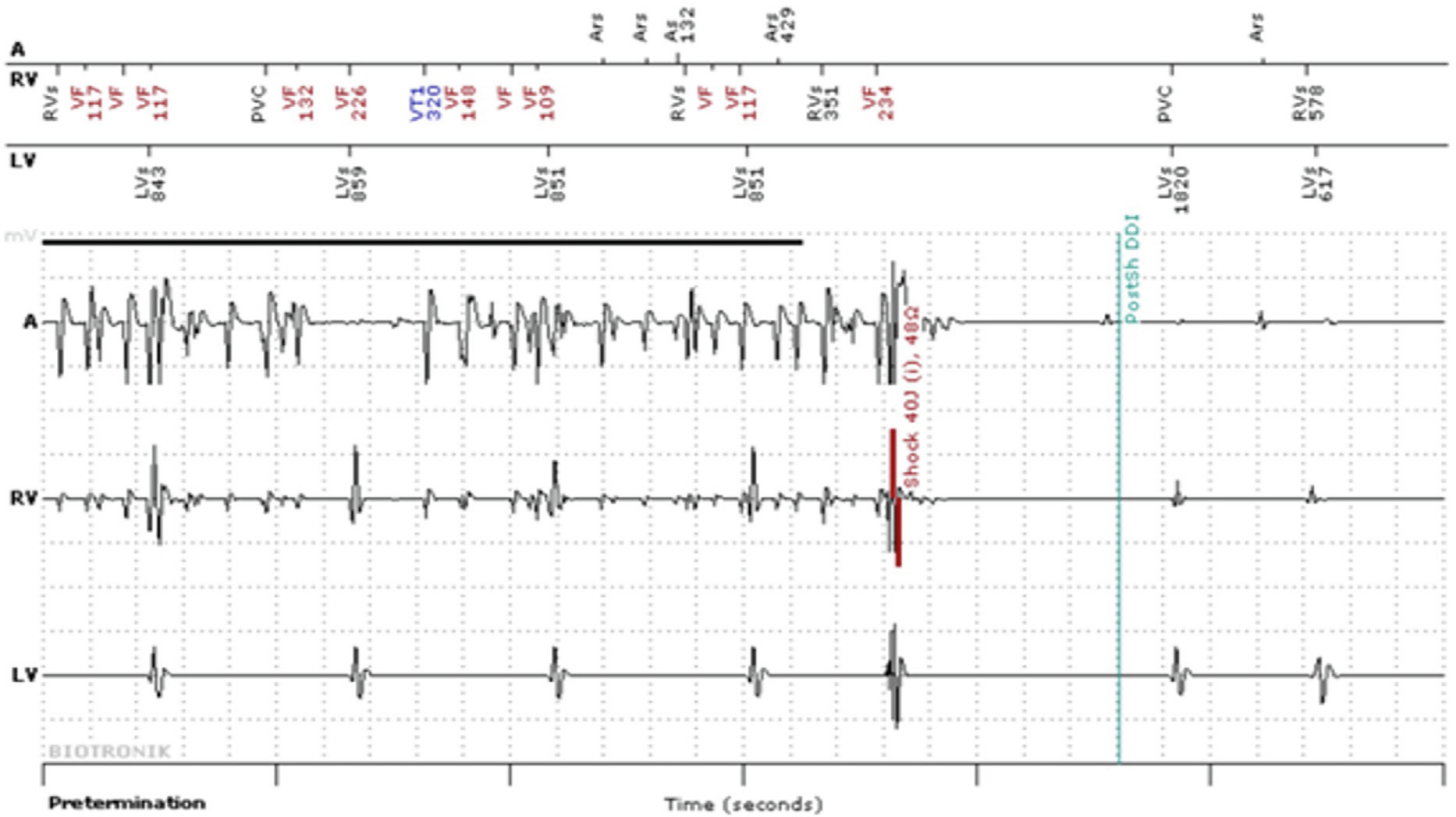
This patient had inappropriate shock during the electroacupuncture session, causing noise on the atrial and ventricular channels, when inadvertently used. This resulted in a remote monitoring alert.

The possibility of early detection of inappropriate therapy, avoiding a sequence of inappropriate shocks or the diagnosis of AF with prompt initiation of anticoagulation, have always been the major benefits of this technology (Figure 2).

The implant-based multiparameter telemonitoring of patients with heart failure (IN-TIME): a randomized controlled trial study demonstrated a reduction in total and cardiovascular mortality in patients with congestive heart failure (CHF) and ICD.^18^ The differences found between the IN-TIME study and others were mainly due to the form of RM used; our study used daily monitoring and therefore identified more events.

A recent meta-analysis demonstrated that early detection of AF reduced stroke incidence.^19^ A meta-analysis published by Hindricks and colleagues^20^ including the efficacy and safety of automatic remote monitoring for implantable cardioverter-defibrillator follow-up: the Lumos-T Safely Reduces Routine Office Device Follow-up (TRUST) trial, a randomized study of remote follow-up of implantable cardioverter defibrillators: safety and efficacy report of the ECOST trial, and IN-TIME studies showed that the daily RM had the greatest benefit in preventing CHF decompensation in patients with reduced EF, findings also observed in our study. This meta-analysis also confirmed a reduction in total mortality or CHF decompensation, as observed in the IN-TIME study. These studies also demonstrated safety and efficacy in the early detection of events in patients with an ICD when compared with the traditional form of follow-up.^21^^,^^22^ Varma and colleagues^21^ demonstrated that adherence to RM was associated with improved survival regardless of cardiac device. RM is also cost-effective.^23^

### Adjusting the Brazilian system to RM

The way the medical service responds to alerts also has a great impact on outcomes; however, this requires adequate organization and resources in these centers to promote effective and immediate follow up.^24^, ^25^, ^26^

Patients with large numbers of events can overload the RM services. It is important to individualize the alert settings to notify the professionals only when necessary. These data were evidenced mainly with the transmissions of implantable loop recorders, devices that were not included in the analysis of our study.^8^^,^^27^ As we had an individualized approach, we did not notice an overload in data analysis in our group. Implantable loop recorders can generate high numbers of alerts and false positive transmissions.

Some centers worldwide have followed the international guidelines, allowing the annual in clinic follow-up of CIED for populations followed by RM, provided that some steps are observed and that patients have access in their regions to in clinic clinical follow-up.^2^, ^3^, ^4^^,^^28^^,^^29^

It would be feasible to create regional centers to analyze the data of large areas of remote monitoring in Brazil that would likely reduce the in-office visits and the overload of the public and private health systems. For a broader implementation of the RM, public services need local centers with structured guidance to receive information and define a plan, especially for red alert scenarios.

### RM and cost reduction

Brazil currently uses about 4% of the gross domestic product in its public health system; studies have shown that this is far below that which is required to provide universal care. The expenses in the private health system were over 9% of the Brazilian gross domestic product in 2017.^13^ Strategies to reduce costs are mandatory to maintain both systems.

RM could bring benefits in terms of cost reduction. This argument is also used by some countries such as Canada, Portugal, and the United States to expand follow-up by RM.^24^^,^^30^ RM published data in South America are scarce, and this study provides relevant information about the use of this form of follow-up in middle-income countries, thus contributing to the debate between medical societies and governments and allowing more patients to have in-home monitoring, especially high-risk groups.^16^

An Italian group published a document in 2020 with the aim of expanding and cooperating centers for the use of MR. ^26^ The Brazilian telemonitoring guidelines classified the RM of CIED as class IIa, considering benefits in cost reduction and early detection of events.^17^ In the American guidelines published in 2015, RM should be offered to all patients with CIED as an additional form of follow-up; this is a class I recommendation.^3^

The psychological aspects of safety and confidence of providers and patients indicate another reason for expanding the use of RM. We verified a high acceptance of this technology with professionals and patients.^31^

### Limitations

This was a single-center study and was unblinded. Patients were followed by the same group that implanted the devices, so we had a greater chance of obtaining positive results, which may not be reproducible in other centers.

## Conclusions

RM proved to be effective in the follow-up of patients with CIEDs, allowing early or elective interventions, which facilitated the therapeutic management of patients. The groups with CRT or CRT-D, reduced EF, and more advanced NYHA functional class had higher event rates. The detection of important alerts was associated with changes in elective or immediate medical treatment. RM should be considered as an additional form of follow-up for patients with CIED in Brazil.
